# Social identity formation among Pride parade participants

**DOI:** 10.3389/fsoc.2026.1719108

**Published:** 2026-04-16

**Authors:** Liya Ai, Chikako Tanimoto

**Affiliations:** Department of Gender Studies, Graduate School of Humanities, Nagoya University, Nagoya, Japan

**Keywords:** ally, LGBT+, Pride march, social identity, social movement

## Abstract

**Introduction:**

This study examines the formation of social identity among Pride parade participants.

**Methods:**

Semi-structured interviews were conducted with 36 individuals who participated in Pride parades in Japan, including 24 identified as LGBT+, 9 as non-LGBT+, and 3 as unspecified. Data were analyzed using constructivist grounded theory.

**Findings:**

The process of social identity formation was categorized into four stages: (1) Belonging to the In-Group, (2) Behaving as an In-Group Member, (3) Recognizing One’s Role within the In-Group, and (4) Universalizing the In-Group Identity. In the Belonging stage, participants perceived themselves as the in-group while positioning heteronormative society as the out-group. In the Behaving stage, shared practices such as carrying rainbow flags and chanting reinforced group solidarity. In the Recognizing stage, participants were differentiated into Core and Supporting roles, highlighting internal role differentiation. In the Universalizing stage, participants emphasized attitudinal differences between in-group and out-group while simultaneously expressing a goal of dissolving such boundaries.

**Discussion:**

These findings contribute to the application of social identity theory to Pride parade participation by demonstrating how collective action fosters shared identity formation among both LGBT+ and non-LGBT+ participants. The study also identifies a novel in-group–out-group configuration, conceptualized as the in-group within the out-group, and suggests the potential for integrating queer theory, particularly the concept of performativity, with social identity theory.

## 1 Introduction

Nowadays, Pride parades often promote diversity and inclusivity, attracting a wide range of participants. As events associated with the LGBT+ (lesbian, gay, bisexual, transgender, and other sexual and gender minorities) community, many participants self-identify as LGBT+. Many of these participants have experienced marginalization in their daily lives due to their sexual orientation or gender identity, often resulting in feelings of loneliness (Elmer et al., 2022). Therefore, it is important for them to have a place where they can connect with other LGBT+ peers.

Previous studies indicate that connecting with other LGBT+ individuals is one of the key motivations for participating in LGBT+ events (Browne, 2007; McFarland, 2012). Pride parades offer opportunities to build these connections and foster a sense of belonging (Hahm et al., 2018). This sense of belonging arises from being in a space surrounded by individuals who share similar sexual orientations or gender identities, experiences, and values, which helps participants feel included and accepted. In particular, shared values, such as advocating for LGBT+ individuals and communities, asserting their presence, and raising their voices for civil rights, help reinforce this sense of belonging regardless of the participants’ own sexual orientations or gender identities. These shared values play a crucial role in forming an in-group identity.

While Pride parades are events primarily for LGBT+ individuals, non-LGBT+ individuals who support LGBT+ also participate. These non-LGBT+ individuals, often referred to as “allies,” are usually defined as members of majority group who support and advocate for oppressed populations (Washington and Evans, 1991). Like LGBT+ participants, non-LGBT+ participants share similar values, such as supporting LGBT+ individuals and communities, increasing their visibility, and advocating for equal rights. Through these shared values, allies may also develop a sense of belonging within Pride parade communities.

Although both LGBT+ and non-LGBT+ participants support LGBT+ rights and share similar values, they are rarely studied as a unified group. Even when attending the same event, prior research on Pride parade participants tends to divide LGBT+ and non-LGBT+ individuals into two distinct groups based on sexual orientation or gender identity (Wahlström et al., 2018). Studies focusing on LGBT+ participants emphasize how Pride events enhances their sense of belonging and connection to others (Tinlin-Dixon et al., 2024; Hahm et al., 2018). In contrast, research on non-LGBT+ individuals has primarily examined their perceptions of Pride parades from the perspective of spectators rather than participants (Mason and Lo, 2009; Tomsen and Markwell, 2009). However, this approach is inconsistent with the inclusive principle of Pride events, which welcome anyone who supports LGBT+ individuals and communities.

In other forms of protest, participants who share a common goal are regarded as members of the same in-group. For example, in environmental protests, participants are viewed as part of a united collective despite differences in age or gender (Fielding et al., 2008). Applying this perspective to Pride parades allows for the formation of a new in-group, “Pride parade participants,” which transcends the boundary between LGBT+ and non-LGBT+ individuals. As members of this in-group, participants share a common identity as LGBT+ supporters, adopting the shared values and behaviors associated with Pride parade participation. This in-group identity is reinforced through comparisons with out-groups, the promotion of in-group favoritism, and repeated participation in parades, whether by attending the same events annually in a single location or by joining different parades across various locations each year. This in-group identity contributes to the construction of their social identity, which can be considered a significant factor for participants in Pride parades.

This study explores the formation of social identity among Pride parade participants, regardless of whether they identify as LGBT+ or non-LGBT+. Specifically, it examines how participants strengthen in-group solidarity and emphasize distinctions between the in-group and out-group. The study investigates how participation in Pride parades contributes to the construction of participants’ social identity. Unlike previous research, which has primarily focused on LGBT+ individuals, this study includes non-LGBT+ allies within its scope. To achieve this, social identity theory (Tajfel, 1970, 1979; Tajfel and Turner, 2004), and identity theory (Stets and Burke, 2000) are employed as the theoretical framework.

## 2 Conceptual framework

Social identity theory proposes that individuals derive a sense of self and belonging from their membership in social groups, explaining how they categorize themselves as part of an in-group distinct from an out-group (Tajfel, 1970). The process of forming group-based identities involves three key stages: social categorization, social identification, and social comparison (Tajfel, 1979). Social categorization is the process through which individuals classify themselves and others into different social groups based on attributes such as age, race, or gender. This process divides people into the in-group “we” (the group they belong to), and the out-group “they” (groups they do not belong to) (Tajfel, 1979). Social identification occurs when individuals adopt the identity of their in-group, internalizing its norms, values, and behaviors. This identification can impact self-esteem by enhancing personal self-worth through a positive sense of belonging to the in-group. Finally, social comparison involves evaluating one’s in-group in relation to relevant out-groups. This process often leads to in-group favoritism and out-group discrimination, as individuals tend to view their own group more favorably than others (Tajfel and Turner, 2004).

However, because social identity is based on group membership, individuals tend to perceive themselves primarily as members of a group rather than as unique individuals (Brewer and Gardner, 1996). As a result, social identity is useful for examining group behavior and intergroup relations but pay less attention to differences among individuals within a group or to the extent to which individuals feel attached to the group. At this point, role identity becomes useful. Unlike social identity theory, identity theory focuses on the roles individuals occupy within a group and the meanings attached to those roles, therefore, paying more attention to intragroup relations and differences among group members (Stets and Burke, 2000). By combining social identity theory and identity theory, it becomes possible to examine not only how social identity is constructed at the group level but also how it emerges through individuals’ roles and interactions within the group. This integrated perspective allows for a more detailed understanding of the process through which social identity is constructed and practiced.

There is a substantial body of research that utilized social identity theory to explore various themes within the LGBT+ community. These themes include workplace experiences (Melton and Cunningham, 2014), mental health service use (Moore et al., 2021), sexual identity formation (Ciszek, 2017), identity management (Aybar Camposano et al., 2022), and identity threat (Minton et al., 2017; Fingerhut and Abdou, 2017). Social identity theory has also been applied in studies focused on LGBT+ events. For example, Ong et al. (2025) found that LGBT+ events foster a sense of affinity with LGBTQ+ communities, helping individuals build confidence in both their personal and social identities. Similarly, Krane et al. (2002) showed that participation in the Gay Games enhanced participants’ social identity by providing a supportive and inclusive environment, fostering greater pride and commitment to the LGBT community, and improving both self-esteem and collective esteem. Furthermore, Hahm et al. (2018) indicated that the sense of belonging experienced by LGBT attendees at LGBT events was associated with event satisfaction, which in turn was strongly linked to their intention to re-attend.

A common thread among these studies is their exclusive focus on LGBT+ individuals, which renders the presence of non-LGBT+ allies at LGBT+ events invisible. To meaningfully discuss the social identity of Pride parade participants, it is necessary to include both LGBT+ and non-LGBT+ participants within the same in-group. In contrast to research on LGBT+ events, which tends to emphasize participants’ insider status as sexual and/or gender minorities, studies of other types of social movements and events often highlight participants’ engagement regardless of their personal identity. For instance, while some protests attract participants because of their connection to specific categories such as age, ethnicity, and occupation (Klandermans, 2002), others, such as environmental protests, draw participants without requiring membership in a particular demographic group. Fielding et al. (2008) demonstrated the role of social identity in shaping participants’ behaviors in these contexts. They found that membership in an environmental organization significantly predicted intentions to engage in environmental activism. Moreover, individuals with more positive attitudes toward environmental activism and stronger perceptions of normative support were more likely to intend to participate in such activities (Fielding et al., 2008). These findings suggest that social identity is not necessarily tied to demographic characteristics but can instead be formed through shared ideology.

Participants’ social identity develops through their involvement in shared social movements. Just as individuals can participate in environmental protests regardless of ethnicity or gender, Pride parade participants are not limited by identity categories as long as they share an interest in LGBT+ issues and a desire to bring about social change for LGBT+ individuals. By participating in Pride parades, individuals engage in collective actions such as marching in public space, expressing solidarity, and advocating social change. Through these practices, participants gradually construct what can be described as an emergent activist identity, which constitutes forms of activism commonly recognized in the international literature on social movements.

In the Japanese context, however, many participants do not explicitly describe themselves as activists. Pride parades are often perceived less as political protests and more as social or celebratory events. Nevertheless, participants’ practices such as marching in public space, demonstrating solidarity, and advocating social change, constitutes forms of activism commonly recognized internationally. Thus, an activist identity may emerge through participation even when participants themselves do not explicitly claim such a label.

Participation in Pride parades can therefore serve as a point of engagement with activism through which both LGBT+ individuals and non-LGBT allies begin to develop an emergent activist identity. At the same time, however, LGBT+ individuals and non-LGBT allies may follow different pathways in arriving at this identity. Accordingly, this study addresses the following research question: How do Pride parade participants construct an emergent activist identity from the perspectives of both LGBT+ and non-LGBT participants? Constructivist grounded theory (Charmaz, 2014) was adopted for data analysis.

## 3 Method

To address the research question, “How do Pride parade participants construct an emergent activist identity from the perspectives of both LGBT+ and non-LGBT+ participants?” this study adopted a qualitative approach, which is well suited for exploring personal experiences and perspectives (Hammarberg et al., 2016). Semi-structured interviews were used because they allow researchers to explore social and personal matters in depth (DiCicco-Bloom and Crabtree, 2006) and give participants the opportunity to express themselves in their own words. Additionally, the interviewer can adjust the order of the questions and introduce new ones on the basis of the participants’ responses. All the interviews in this study were conducted and transcribed in Japanese. Interviews were conducted either in person or online. During the preparation of this manuscript, the authors used ChatGPT-5 to proofread and improve the clarity of the English language. This study is part of a larger project that investigates various aspects of participation in Pride parades, and it draws on the same set of interview data as other manuscripts but applies a different analytical lens focusing on social identity.

### 3.1 Participants

Data were collected in 2023 (*n* = 25) and 2024 (*n* = 11) using semi-structured interviews conducted either in person or online. The interviews were scheduled between one and 3 months after the events, depending on the participants’ availability and preferences. The interviews ranged from 25 to 90 min in length. All interviews were recorded. Participants were recruited by the first author through in-person outreach at Pride parade venues and LGBT+ social events, as well as online via social media and online social events. In-person recruitment took place after Pride parades. For online events, organizer permission was obtained beforehand, and recruitment was conducted during the final segment of the event. Snowball sampling was also employed to reach friends and colleagues with whom participants attended the parades. The inclusion criteria required participants to be at least 18 years old and to have participated in a Pride parade in 2023 or 2024.

A total of 36 participants took part in this study, including 24 who identified as LGBT+, nine as non-LGBT+ (heterosexual and cisgender), and three as unspecified. The unspecified group consisted of individuals whose gender identity was “woman”; two were unsure of their sexual orientation, and one did not respond to the sexual orientation question. Any participant who self-identified having a non-heterosexual and/or non-cisgender identity was categorized as LGBT+, even if additional uncertainty was expressed. Individuals who explicitly identified as both cisgender and heterosexual were classified as non-LGBT+. In terms of gender identity, the sample included 25 cisgender individuals, four transgender individuals, and seven individuals categorized as “other”: one was not sure, one was undecided, one identified as non-binary, one as MtQ, two as MtX (with X indicating individuals who do not identify strictly as male or female), and one as FtX. With respect to sexual orientation, the sample included seven gay individuals, three lesbians (including one who expressed uncertainty), two bisexuals, three asexuals, three pansexuals, twelve heterosexuals, and six categorized as “other”: two did not answer, one identified as “Fluid,” two were “Not Sure,” and one identified as “Heterosexual but possibly Pansexual.”

In terms of nationality, the sample included thirty-one Japanese participants, three Chinese participants, one American participant, and one participant who did not respond; all lived in Japan. Participants ranged in age from 20 to 62 years (*M* = 33, SD = 11.80). Table 1 presents participants’ sexual minority status and interview mode.

**Table 1:** Participants’ information (*N* = 36).

Category	*n*	%
Sexual minority status		
LGBT+	24	66.7
Non-LGBT+	9	25.0
Unspecified	3	8.3
Interview mode		
Online	32	88.9
In-person	4	11.1

### 3.2 Data collection

Participants were asked a series of semi-structured questions about their experiences of participating in Pride parades, their motivations for participation, their personal meanings attached to Pride parades, and their views on the role of Pride parades in society. The interviews were conducted at different points between May 2023 and July 2024. Most interviews were conducted online (*n* = 32), while a smaller number were conducted in person (*n* = 4). Online interviews were conducted via Zoom, while in-person interviews were conducted in cafés and on university campuses. Examples of interview questions included:

What motivated you to take part in the Pride parade?
What does the Pride parade mean to you personally?
Do you think Pride parades are important for LGBT+ people in Japan? Why or why not?
What message would you like to convey through the Pride parade, and who would you like it to reach?

Follow-up questions were asked to explore participants’ experiences and perspectives in greater depth.

### 3.3 Ethical considerations

All participants provided informed consent prior to participation. Written consent was obtained either through signed paper forms or electronic documents. The consent process covered the purpose of the study, procedures, voluntary participation (including the right to withdrawal at any time without penalty), recording, and the use of data in translation and publication. Participants were informed that the study had received institutional ethics approval before taking part. The research was reviewed and approved by the Ethics Committee at Nagoya University (Approval No. NUHM-22-009). Pseudonyms were used to protect participant confidentiality.

### 3.4 Data analysis

The data were analyzed using constructivist grounded theory (CGT), developed by Charmaz. CGT builds on the grounded theory approach originally developed by Glaser and Strauss (1967) but emphasizes the co-construction of data and theory between researchers and participants (Charmaz, 2014). Following the CGT coding process, the transcripts were analyzed line by line to develop distinct initial codes. During this phase, memo-writing was used to capture key concepts and emerging ideas. These initial codes were then reviewed and refined into focused codes on the basis of their frequency and significance. Broader categories were subsequently developed from the focused codes. Links between these categories were established, and theoretical insights were constructed by incorporating reflections and patterns from the memo-writing process. This process was repeated with each round of interview data collection until theoretical saturation was reached—that is, the point at which no new codes or categories emerged from the data. When presenting interview quotations, participants’ sexual orientation and gender identity are indicated in parentheses, for example (Gay, Man). Table 2 presents an example of the data analysis.

**Table 2:** An example of the constructivist grounded theory analysis process.

Data	Initial coding	Focused coding
I felt an overwhelming sense of joy [when I marched in the Pride parade]. For example, even if I talked to a stranger marching next to me, we could have a very friendly conversation. This was possible because, even without knowing each other, we shared a common experience of marching in the parade. That made me feel that I had companions, and that feeling was really encouraging.	Sense of joy during the march Friendly conversation with strangers Shared experience with strangers Feeling encouraged by companions	Marching as a shared experience with strangers made the participant feel a sense of companionship

## 4 Findings

**Figure 1:**
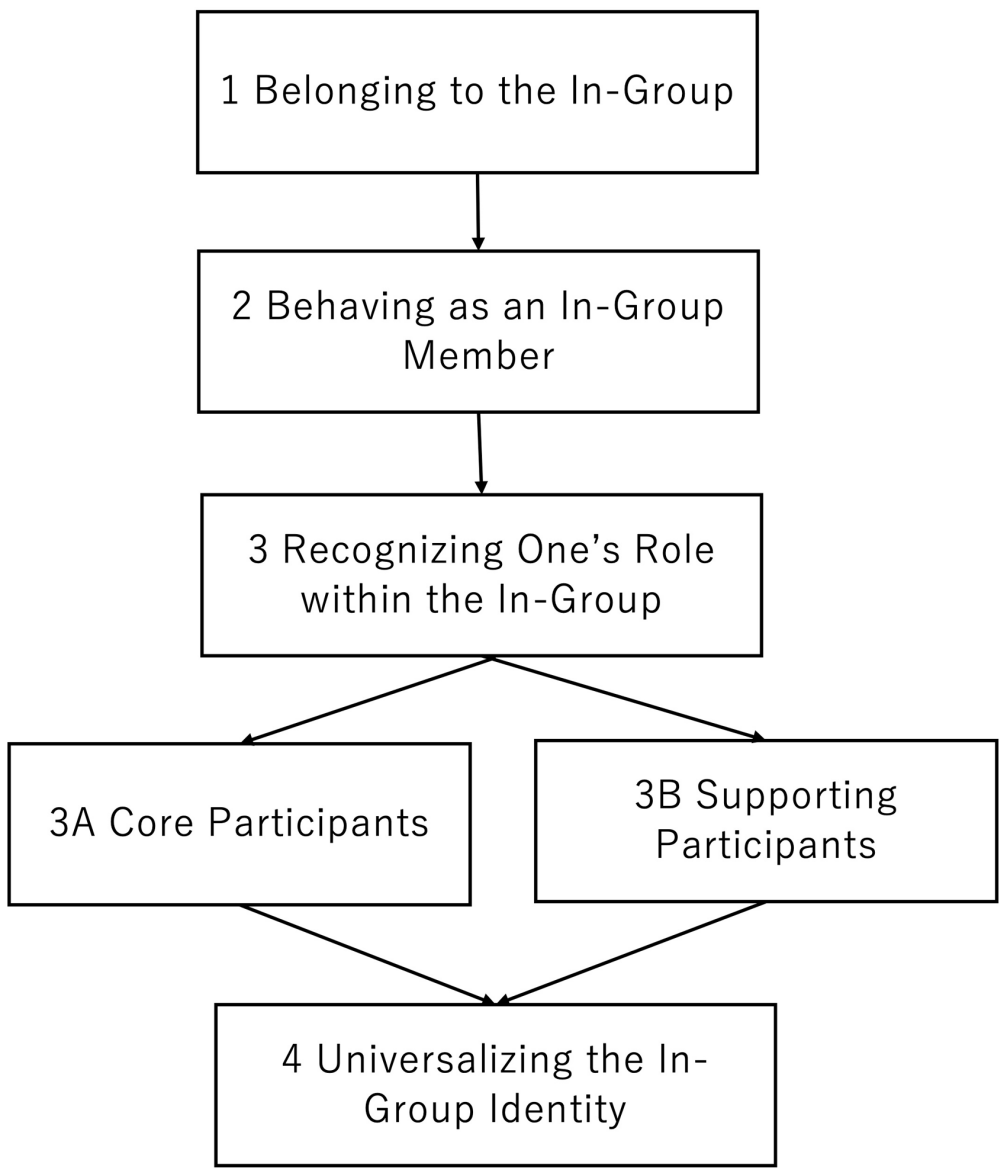
Formation of emergent activist identity among pride parade participants.

Regarding the research question “How do Pride parade participants construct an emergent activist identity from the perspectives of both LGBT+ and non-LGBT+ participants?”, four stages were identified from the data: (1) Belonging to the In-Group, (2) Behaving as an In-Group Member, (3) Recognizing One’s Role within the In-Group, and (4) Universalizing the In-Group Identity. The third stage, Recognizing One’s Role within the In-Group, consists of two subcategories: (3A) Core Participants and (3B) Supporting Participants. Figure 1 illustrates the process of emergent activist identity construction.

### 4.1 Belonging to the in-group

The first step of social identity formation is social categorization, in which individuals categorize themselves and others into social groups. Pride parade participants categorized themselves as members of an in-group and experienced a sense of belonging within this category. As in-group members, both LGBT+ and non-LGBT+ participants shared common values as LGBT+ supporters and categorized all parade participants as companions, regardless of their sexual orientation or gender identity. Through this process, they categorized the out-group as the heteronormative society consisting of individuals indifferent to LGBT+ issues and the broader social structure and distinguished themselves from it.

The data suggests that individuals who participated in Pride parades can be defined as an in-group. This categorization is based on participants’ perceptions of their fellow marchers as in-group members and the anticipated audiences as the out-group. The in-group includes both LGBT+ and non-LGBT+ individuals who share a common direction and purpose. By marching together, participants strengthen their sense of unity. For example, Sora (Heterosexual, Trans woman) stated “I think there is *a strong sense of unity* here, showing that the people *marching together* are not alone—they are part of a group.” Similarly, Oto (Heterosexual, Trans Woman) noted, “While marching, I really felt *a strong sense of unity* with the others I marched alongside.”

Non-LGBT+ participants also valued the experience of walking together. Satoshi (Heterosexual, Man) explained:

It felt like I was part of it, not just being there, but really engaging with *a sense of unity*. In a way, I felt like I became part of it, like I was one of the people directly involved. I was able to share a little bit of the feeling with that large gathering.

Similarly, Mei (Heterosexual, Woman) remarked, “It was fun to be able to *walk together* with LGBTQ people in the same direction and make a statement together for everyone to see.”

Both LGBT+ and non-LGBT+ participants emphasized that they share the same political goals and values, viewing the Pride parade as an opportunity to raise their voices. Participants frequently used pronouns such as “we” to emphasize their membership in the same group. For instance, Riku (Gay, Man) shared: “*We* must speak up because without doing so, there is no social support. […] Nothing will change unless *we* keep raising *our* voices. […] By speaking up, *we* are pushing for change.” Non-LGBT+ participants expressed comparable views. Mei (Heterosexual, Woman) noted, “I think Pride parade is a great opportunity to let many people know what *we* want them to understand. […] It’s an open space where *we* can reach out to people directly.” These statements indicate that Pride parade participants formed an in-group by categorizing themselves as in-group members and using inclusive languages such as “we” to emphasize their sense of belonging. Additionally, they viewed the Pride parade as an opportunity to raise awareness about LGBT+ individuals and issues.

The data show that both LGBT+ and non-LGBT+ participants considered the broader heteronormative society as an out-group. Notably, they did not identify any specific group as the out-group; instead, they envisioned a loosely defined and fragmented out-group that was not clearly categorized. The out-group includes not only those who discriminate against LGBT+ individuals but also those who are indifferent to LGBT+ issues. For instance, Mizuki (Pansexual, Not Decided) stated, “I want to tell those who know nothing [about LGBT+ issues], or those who think they don’t know anyone [who is LGBT+], that we actually exist nearby.” Non-LGBT+ participants also had similar statements. Saki (Heterosexual, Woman) remarked that she wanted the general public, particularly middle aged and older people, to become more aware of LGBT+ issues. It is notable that participants consistently used the third person plural “they” to refer to the out-group members. Participants viewed their march through the street as a way to appeal to these people.

The social structure is another element that constructs the out-group. Research participants frequently mentioned terms such as society, social system, and politics. Sora (Heterosexual, Trans Woman) noted, “I’m participating in the parade with a strong desire to send a message to society.” Similarly, Nagi (Heterosexual, Trans Man) stated, “A parade is essentially a form of protest. It’s treated as a protest, so the police are there for security, and permits are required. But it’s also a way to make an appeal to the nation.” Non-LGBT+ participants also emphasized the importance of sending a message to society.

Some described the Pride parade as a form of protest directed at a society that marginalizes LGBT+ individuals. Mainstream society was seen as one that recognizes only heterosexual and cisgender identities as “normal,” excluding those who deviate from this norm. The out-group was portrayed as something larger, encapsulated by the concept of normalcy. Meanwhile, the in-group was described as those who do not conform to this normalcy. Tomoya (Heterosexual, Man) noted, “A society and social systems primarily organized around cisgender heterosexual men—that is what some people consider ‘normal.’ So it’s not really about any specific attribute, but rather what is commonly referred to as ‘normal’ in society.”

### 4.2 Behaving as an in-group member

Behaving as an in-group member represents the second stage in the formation of social identity for Pride parade participants. This stage corresponds to the process of social identification, in which individuals come to define themselves as members of the group. Participants express this identification through their appearance and behavior, thereby enacting their in-group identity in visible and performative ways.

Appearance is one of the primary means by which participants display their in-group identification. Almost all participants mentioned that they held rainbow flags during the march. The rainbow, as a symbol of the LGBT+ community, is widely used to signify either membership in or support for the community. Holding rainbow items during the Pride march gave participants a sense of unity with those walking alongside them. As Oto (Heterosexual, Trans Woman) remarked, “In everyday life, you often can’t tell if someone is gay or lesbian just by looking at them. That’s why I think walking in the parade and expressing one’s sexuality through clothing is really important.” Some participants wore rainbow-colored clothing during the parades. For example, Kayo (Lesbian/Not Sure, Woman) noted, “I wore a rainbow outfit that I had, attached a rainbow badge to my bag, and then wrapped a large rainbow flag around myself when I marched.” Similarly, Satoshi (Heterosexual, Man) said, “While I was at the event, I had a big rainbow-patterned cloth around my waist […] to show solidarity [with the LGBT+ community].”

Another way participants demonstrate in-group identification was through their behavior. The most noticeable example was marching together in the parade. Another common behavior was chanting “Happy Pride” while waving to the audience. Satoshi (Heterosexual, Man) explained:

You get to interact with strangers without needing to exchange contact information. It’s not like you just walk silently for an hour. Even if you stay quiet, simply saying ‘Happy Pride’ or waving hands together creates a sense of connection.

Similarly, Rei (Not Sure, Woman) noted, “Even though we don’t know each other very well, we waved rainbow items at each other to show that we were [buddies], and the ‘Happy Pride’ calls came naturally from all around.”

Almost all Pride parade participants shared a desire to make society more inclusive for LGBT+ individuals and aimed to encourage them through visible actions. By carrying or wearing rainbow items during the Pride parades, participants affirmed the presence of companions and reinforced their in-group identity. Moreover, through shared actions such as calling “Happy Pride” and waving, they created a visible community, where LGBT+ and non-LGBT+ participants came together and show their support for LGBT+ individuals. Notably, Pride parades offer non-LGBT+ participants a unique opportunity to temporarily bridge the gap between themselves and LGBT+ participants.

### 4.3 Recognizing one’s role within the in-group

Recognizing one’s role within the in-group indicates the third stage in the formation of social identity as Pride parade participants. Although both LGBT+-identified and non-LGBT+ parade participants are members of the in-group, their statuses differ. As a result, LGBT+ participants and non-LGBT+ participants play different roles within the in-group: LGBT+ participants as core participants and non-LGBT+ participants as supporting participants.

#### 4.3.1 Core participants

Individuals who identify as LGBT+ inherently belong to the in-group from the outset because they experience unequal treatment in a heteronormative and cisnormative society. Their participation was grounded in their positionality as LGBT+ individuals, and they can therefore be understood as core participants in Pride parades. Honoka (Lesbian, Woman) noted that through participating in parades, she as a member of the LGBT+ community can contribute to the visibility of LGBT+ individuals. She also stated, “If there is no [Pride parade], we will be treated as not existing, especially in regional areas, so I think Pride parades are necessary events.” Similarly, Haru (Asexual, Not Sure) said, “Because I’m a sexual minority, I felt a desire to take action to change my life.”

Some expressed a sense of commitment to participating in Pride parades as a way to raise their voices. For example, Natsuki (Panromantic Asexual, X-gender) noted that they participated because they are part of the LGBT+ community and felt that they played a central role. Similarly, Nao (Asexual, Q) stated, “There are messages that only LGBT+ individuals can convey.” For instance, Sora (Heterosexual, Trans Woman) remarked:

I think it’s important to show through the parade that there are many [LGBT+] people in society and to make people aware of it. I hope it encourages people to think that some of their friends or family members might also be LGBT+. I also hope it can become a catalyst for changing the country’s legal system.

Other participants referred to their LGBT+-friends’ experiences as encouraging them to participate in parades. Nagi (Heterosexual, Trans Man) said that he has friends living in regional areas who cannot come out. He noted, “Since there are people [in our community] who cannot raise their voices, I, as someone who can speak up, feel that I need to raise my voice to change society.” These perspectives are rooted in the experiences of LGBT+ individuals. Because they have experienced unequal treatment or difficulties by themselves or have heard about such experiences from their friends in their daily lives, they develop a sense of duty to raise their voices; otherwise, the situation will not improve. Pride parades, as public events, provides opportunities for them to do so. As LGBT+ individuals themselves, they position themselves as core participants in Pride parades while also speaking on behalf of others who cannot participate.

#### 4.3.2 Supporting participants

In contrast, non-LGBT+ allies tend to hold different perspectives as their participation is more strongly motivated by political considerations or a sense of justice. Being non-LGBT+ positioned them as supporting participants in Pride parades. For instance, Tomoya (Heterosexual, Man) stated that participating in Pride parades made him recognize his privilege as a heterosexual man. Another ally participant, Wakana (Heterosexual, Woman), remarked that attending the Pride event provided her with a valuable experience of being in a minority position. In a heteronormative society, she, as a heterosexual woman, belongs to the majority, whereas LGBT+ individuals are the minority. However, at the Pride event, which centers on LGBT+ communities, LGBT+ individuals form the majority, and she was able to experience what it feels like to be the minority. These perspectives show that although non-LGBT+ participants were members of Pride parades, they still recognized that their positions differed from those of LGBT+ participants. This difference in position within the same in-group led them to feel that they needed to be accepted as members of the group. For example, Miki (Heterosexual, Woman) stated that, “When I called out to others and exchanged high-fives with strangers, it made me really happy. It felt like I had been accepted as part of the group.” Similarly, Mei (Heterosexual, Woman) said that she enjoyed marching with LGBT+ people and reflected on the experience: “It made me feel that I want to support them, and it also became an opportunity for me to think about what I might be able to do in the future.” These statements indicate that non-LGBT+ participants played a supporting role in Pride parades due to their different status.

### 4.4 Universalizing the in-group identity

Universalizing the in-group identity represents the final stage in the formation of social identity among Pride parade participants. This stage corresponds to the process of social comparison, in which participants interpret the relationship between the in-group and the broader society. In the universalizing view, all humans are regarded as fundamentally the same. Participants perceive in-group members as sharing this universalizing perspective, according to which there is no essential difference between LGBT+ and non-LGBT individuals. By adopting this view, the role distinctions that emerged in the previous stage are symbolically dissolved, thereby reinforcing a shared in-group identity. At the same time, participants perceive the outside world as operating through a minoritizing perspective in which LGBT+ individuals are considered as different from others and consequently positioned as marginalized.

From the data, both LGBT+ and non-LGBT+ Pride parade participants emphasized that LGBT+ individuals are fundamentally the same as the heterosexual majority; everyone has the rights to live their own life. For example, Mei (Heterosexual, Woman) remarked, “I want to convey to everyone that we all have the same rights and that we are all the same. I want to share this message with as many people as possible.” From her perspective as a heterosexual participant, she emphasized there is no significant difference between LGBT+ and non-LGBT+ individuals. In other words, she participated in the Pride parade to raise awareness of this similarity. LGBT+ participants also expressed similar views. Mana (Bisexual, Woman) stated:

I’d like people to stop thinking it’s weird that I live with a same-sex partner, or that I do not get married but live with a woman. I’m no different from anyone else just living their life normally. It’s not like I’ll suddenly eat something bizarre. What I’m advocating for is simply to be treated as normal.

Similarly, Nao (Asexual, Q) noted, “We’re not special or different. We’re just ordinary people who are around you. We shouldn’t be seen as pitiful or treated differently. […] Gender is simply one of the natural traits people have.”

These statements suggest that LGBT+ individuals do not seek different treatment but rather wish to be regarded as equal to the heterosexual majority. At the same time, non-LGBT+ participants recognize the similarities between LGBT+ and non-LGBT+ individuals. This sense of similarity and shared values not only motivated participation in the parades but also contributed to the formation of a shared social identity as Pride parade participants.

In contrast, participants perceived the out-group as holding a minoritizing view: one that marks minorities as distinct from and subordinate to the majority. Chihiro (Lesbian, Trans Woman) stated that laws related to sexual minorities are created by the majority, resulting in various restrictions on LGBT+ individuals. This statement suggests that pride parade participants, particularly those who identify as LGBT+, are critical of majority privileges and the unequal treatment that minorities experience in society. On the other hand, however, participants viewed the use of terms such as “majorities” and “minorities” as problematic because these terms reinforce binary distinctions. For example, Hikari (Pansexual, Non-binary) remarked, “I sometimes question whether grouping people under the umbrella term ‘LGBTQ’ has inherent value. Although categorizing people can aid understanding, it may also contribute to social division.” Similarly, Sora (Heterosexual, Trans Woman) noted, “I believe breaking down the societal barriers created by words like ‘minority’ and ‘majority’ is the greatest challenge we must overcome.” They highlighted that labels such as “majority” and “minority” contribute to unfairness by categorizing people based on their sexual orientation and gender identity, thereby reinforcing social divides.

Participants considered that out-group members are often unaware of their privileges stemming from this unfair division, as they rarely encounter issues related to their sexual orientation or gender identity. Participants also considered that this lack of awareness highlights a significant difference between in-group and out-group members. Ironically, however, by constructing the out-group in this way, participants inadvertently reinforced the boundary between the two, thereby positioning the in-group itself in opposition to the very differentiation it seeks to overcome.

## 5 Discussion

The findings from the data indicate that Pride parade participants viewed themselves as part of an in-group of LGBT+ supporters and enhanced their solidarity with other group members through similar appearances and behaviors, regardless of sexual orientation or gender identity. They drew a clear line between the in-group to which they belonged and the out-group, which included individuals indifferent to LGBT+ issues and to social structure more broadly. These findings align with the processes outlined in social identity theory: Social Categorization, Social Identification, and Social Comparison. At the same time, the data show role differentiation within the in-group, distinguishing LGBT+ individuals and from non-LGBT+ allies. The data also reveal participants’ desire to deconstruct the boundaries both between LGBT+ individuals and non-LGBT+ allies within the in-group and also between the in-group and the out-group. This ambivalence between strengthening group identity and challenging group boundaries captures the complex dynamics of solidarity and inclusion within the Pride movement.

### 5.1 Comparison with social identity theory

According to social identity theory, there is an implicit assumption that specific out-groups exist for in-group members. However, the findings of this study suggest that the definition of out-groups is ambiguous and that no clearly defined out-groups exist. Participants loosely defined the out-group as those absent from Pride parades, while viewing themselves, both LGBT+ and allies, as part of an inclusive in-group. For them, the out-group is not a specific entity. Rather, it encompasses the broader heteronormative society, including both non-LGBT+ individuals who are indifferent to LGBT+ issues and the societal structures that perpetuate inequality. Traditionally, social identity theory conceptualizes in-groups and out-groups as two separate and independent groups, where LGBT+ individuals constitute the in-group and non-LGBT+ individuals are positioned as the out-group (Wahlström et al., 2018). However, the present findings suggest a more nuanced configuration: the in-group exists within the broader out-group, and non-LGBT+ individuals may belong to either group depending on the extent to which they actively support LGBT+ individuals. In interpreting these group boundaries, the analysis relied not only on explicit pronouns, such as “we” and “they,” but also on the broader narrative context of participants’ statements. The diffuse construction of the out-group observed in this study may partly reflect socio-cultural dynamics in Japan, where Pride parades tend to emphasize celebration and awareness-raising rather than direct confrontation with specific groups or institutions. However, the findings also suggest that social movement identities may sometimes construct out-groups not as clearly bounded opposing groups but as broader social structures. In this sense, the results offer insights that may be extended beyond the Japanese context and point to the importance of future comparative research across different cultural settings.

This study also found evidence of in-group favoritism through social comparison between in-group and out-group members. Pride parade participants described the parades as inclusive social movements and positioned their in-group as advocates for inclusivity. Among non-LGBT+ participants in particular, an increase in self-esteem was evident. While acknowledging their privilege as members of the heterosexual majority, they distinguished themselves from out-group non-LGBT+ individuals by demonstrating interest in LGBT+ issues and actively working toward a more inclusive society. This finding is consistent with a meta-analysis of 29 studies showing that high self-esteem is associated with stronger in-group identification (Rivera et al., 2024).

The shared social identity of in-group members in the current study is not based on sexual orientation or gender identity but rather on activist identity. LGBT+ participants were more likely to describe participation in Pride parades as a duty. Many had encountered various forms of difficulty and felt a need to take action to change their circumstances. This motivated them to participate in Pride parade and raise their voices for themselves. At the same time, some participants noted that they also spoke out for other LGBT+ individuals, suggesting that they also understood themselves as supporters of the LGBT+ community. From this perspective, their activist identity appears to develop relatively naturally, often without being fully recognized as such. Consequently, LGBT+ individuals who have not participated in Pride parades may be viewed as potential participants but are not necessarily considered members of the in-group. These findings align with previous research indicating that conviction, a sense of duty, and minority identity motivate individuals to participate in Pride parades (Peterson et al., 2018).

Conversely, non-LGBT+ allies who participate in Pride parades are perceived as in-group members because they share core ideological commitments with LGBT+ participants, such as the belief that LGBT+ individuals should not face discrimination and that heteronormative social structures should change, and because they actively engage in collective action for equality. Russell (2011) found that fundamental principles such as justice, civil rights, religious belief, and awareness of privilege can motivate heterosexual allies to engage in collective action for the rights of LGBT individuals. Similar principles emerged in this study. Moreover, non-LGBT+ participants were more likely to describe themselves as supporters of the LGBT+ community. Because they do not identify as LGBT+, they acknowledged that they may not fully understand the experiences and difficulties faced by LGBT+ individuals. As a result, although non-LGBT+ allies participate in Pride parades and engage in activities similar to those of LGBT+ participants, they tend to perceive their participation as being accepted by the LGBT+ community and permitted to be part of it. In this sense, while LGBT+ participants often develop an activist identity through experience, non-LGBT+ allies tend to construct their activist identity in more explicitly political terms. The role differences between the LGBT+ individuals and non-LGBT+ allies within the in-group suggest that identity theory is useful for explaining relationships within the in group.

Previous research on the relationship between social identity and activism indicates that individuals develop a social identity within specific groups, which leads to participation in social activism. For example, Liss et al. (2004) found that female college students who self-identified as feminists and believed that women should work together to achieve goals were significantly more likely to engage in collective behaviors. Similarly, based on studies of elderly political participation, peaceful protests, and farmers’ demonstrations, Klandermans (2002) found that when individuals identify with a specific group, they are more likely to participate in protests addressing injustices against that group. These findings indicate that individuals often join protests because they already possess a social identity.

However, the findings of this study indicate that the construction of LGBT+ activist identity emerged through participation in Pride parades, rather than pre-existing prior to these events. This suggests that an activist identity, characterized by enthusiastic engagement in Pride parades, is distinct from the identity of simply being LGBT+. This distinction is particularly evident among non-LGBT+ allies, providing insight into how they construct an LGBT+ activist identity through their involvement in Pride parades. Specifically, this process is performative, involving repeated behaviors such as waving, chanting, and calling out “Happy Pride” during the parades. Additionally, repeated actions—such as participating in the same Pride parade annually or attending multiple Pride parades in different locations—further contribute to the construction of an LGBT+ activist identity. This dynamic between identity and action supports the view that identity is performatively constructed (Butler, 1990, 1993). Although the term “activist” may carry different connotations in Japan compared with other counties, the behaviors observed among Pride parade participants can be understood as forms of emergent activism. In this sense, the findings of this study have relevance beyond the Japanese context.

### 5.2 The universalizing view and the minoritizing view

Despite the differences in sexual orientation or gender identity between LGBT+ and non-LGBT+ participants, Pride parade participants shared a universalizing view that emphasized their shared experiences and commonalities. They consistently expressed the belief that LGBT+ individuals are not abnormal but normal, framing in-group members as equally valuable or even morally superior due to their advocacy for universal equality. By aligning themselves with an in-group, both LGBT+ and non-LGBT+ participants embraced a shared ideology within the community. Together, they challenged social norms, stereotypes, and the marginalization of LGBT+ individuals. A key distinction between in-group and out-group members lies in whether they recognize these similarities and acknowledge the inequality inherent in a social system that privileges heterosexuality.

As Sedgwick (1990) argued, the homo/heterosexual distinction can be understood from two perspectives: separatist and integrative. The separatist perspective views gender and sexuality in minoritizing terms, whereas the integrative perspective emphasizes universalization. Pride parade participants in this study emphasized the importance of universalization, under which non-LGBT+ allies who join the parades are recognized as members of the in-group. Without the inclusion of non-LGBT+ allies, the in-group would become a minoritized group, leaving little room for ally engagement. By including non-LGBT+ allies, Pride parades demonstrate the possibility of fostering inclusivity and advancing the universalization of sexual orientation and gender identity. Ultimately, participants aim to bridge the gap not only between the LGBT+ and non-LGBT+ participants within the in-group but also between in-group and out-group members in the broader society.

Nevertheless, in-group members sometimes reinforce their own moral superiority by framing the values of the out-group as minoritizing while positioning their own stance as universalizing. This differentiation reveals a tension within the movement, as the attempt to universalize sexual orientation and gender identity simultaneously relies on distinctions that reproduce group boundaries. This paradox highlights a central tension within Pride participation: the pursuit of universalization often coexists with discursive practices that reproduce distinctions between in-group and out-group members.

### 5.3 Implications

This study makes several theoretical contributions to the understanding of social identity formation in Pride movements. First, it illustrates the process through which Pride parade participants performatively construct an emergent activist identity through their participation in Pride parades. By drawing on social identity theory, the study demonstrates how collective action can foster the development of a shared social identity among Pride parade participants. By incorporating identity theory into social identity theory, this study also highlights role differentiation within the in-group. This theoretical integration enables a more nuanced understanding of the internal dynamics of the movement, particularly the different roles played by LGBT+ individuals and non-LGBT+ allies. While previous studies have examined Pride parade participants as two distinct groups based on sexual orientation or gender identity (Peterson et al., 2018), this study instead conceptualizes LGBT+ individuals and non-LGBT+ allies as members of a collective group who share values and solidarity within the context of Pride parades.

Second, this study advances social identity theory by introducing a new perspective on the definition of the out-group, which differs from the traditional understanding in which the out-group is defined as a group to which in-group members do not belong (Tajfel, 1979). When both LGBT+ and non-LGBT+ Pride parade participants are regarded as part of the in-group, the out-group shifts from a specific set of individuals to broader society. This reconceptualization frames Pride parade participants as the in-group and heteronormative society as the out-group, while highlighting a unique dynamic in which the in-group exists within the out-group. In this sense, the in-group is not positioned as a separate or independent group but as one embedded within the broader out-group.

Third, the study suggests potential directions for the future development of social identity theory. The findings indicate that the concept of performativity from queer theory (Butler, 1990, 1993) can provide deeper insights into the process of social identity formation. For example, performances such as wearing rainbow outfits and chanting “Happy Pride” during parades contribute to the reinforcement of social identity among in-group members. Visual symbols such as rainbow flags, pins, and coordinated clothing function as performative acts through which participants publicly enact and reinforce their belonging to the Pride community. These findings suggest that integrating queer theory with social identity theory may open new avenues for understanding how social identities are not only cognitively formed but also performatively enacted in collective action.

Finally, the findings resonate with Sedgwick’s (1990) distinction between minoritizing and universalizing views of gender and sexuality. Pride parade participants emphasize universalization by including non-LGBT+ allies within the in-group and seeking broader social inclusion. At the same time, however, they often frame heteronormative values as minoritizing, which reveals a tension between the pursuit of universalization and differentiation from the out-group. This paradox suggests that even movements seeking universal inclusion may rely on boundary-making practices that reproduce distinctions between in-group and out-group members.

### 5.4 Limitations

This study has several limitations. First, there are limitations related to language. Because interviews were used as the primary research method, participants’ social identities were inferred partly from their use of pronouns such as “we” or “they” in their statements. However, such expressions were not consistently present in the interview responses. This scarcity may stem from the tendency in Japanese to omit subjects when the context is clear to both the speaker and the listener. Given that language and culture can influence the expression and interpretation of social identity, future research on Pride parade participants should more systematically consider the role of language.

Second, the meaning associated with the term “activist” may differ across cultural contexts. In Japan, the word often carries a relatively negative connotation, which may influence how participants perceive activism and whether they identify themselves using this label. In this study, however, the concept of “activist” identity is used as an analytical term grounded in international academic discourse rather than in the everyday linguistic context of Japan. Future research could therefore examine how the meaning and interpretation of activist identity vary across cultural contexts and how these differences shape participation in Pride movements.

Finally, although this study incorporated identity theory to explain role differentiation within the in-group, these differences were not examined in depth. Future research should explore intragroup variation more fully, particularly how the different identities shape the roles, perspectives, and emotional orientations of Pride parade participants. While social identity theory explains the formation of shared identity, identity theory may be better suited to understanding diversity within the in-group (Stets and Burke, 2000). Future research should further advance the integration of social identity theory and identity theory to provide a more comprehensive framework for understanding both intergroup boundaries and intra-group role differentiation.

## 6 Conclusion

Overall, this study indicates that Pride parade participants have formed a social identity that transcends sexual orientation and gender identity. They reinforced their identity as Pride parade participants—as a shared social identity—through similar appearances and behaviors during the parades as well as a shared belief in inclusion. A significant finding of this study is that both LGBT+ and non-LGBT+ participants perceive themselves as in-group members, while they view heteronormative society, including individuals indifferent to LGBT+ issues and the broader social structure, as the out-group. Although the in-group formed its social identity by distinguishing itself from the heteronormative out-group, its ultimate goal is to dismantle this boundary and cultivate a more inclusive society.

## Data Availability

The datasets presented in this article are not readily available due to confidentiality agreements with participants. Requests to access the datasets should be directed to the corresponding author at lykb1121@gmail.com.
